# Clinical Epidemiology of Dengue and COVID-19 Co-infection Among the Residents in Dhaka, Bangladesh, 2021–2023: A Cross-sectional Study

**DOI:** 10.1093/ofid/ofaf039

**Published:** 2025-01-25

**Authors:** Nadim Sharif, Rubayet Rayhan Opu, Afsana Khan, Tama Saha, Abdullah Ibna Masud, Jannatin Naim, Zaily Leticia Velázquez Martinez, Carlos Osorio García, Meshari A Alsuwat, Fuad M Alzahrani, Khalid J Alzahrani, Isabel De la Torre Díez, Shuvra Kanti Dey

**Affiliations:** Department of Microbiology, Jahangirnagar University, Savar, Dhaka, Bangladesh; Department of Microbiology, Jahangirnagar University, Savar, Dhaka, Bangladesh; Department of Statistics and Data Science, Jahangirnagar University, Savar, Dhaka, Bangladesh; Department of Microbiology, Jahangirnagar University, Savar, Dhaka, Bangladesh; Department of Microbiology, Jahangirnagar University, Savar, Dhaka, Bangladesh; Department of Microbiology, Jahangirnagar University, Savar, Dhaka, Bangladesh; Universidad Europea del Atlántico, Isabel Torres 21 Santander, Spain; Universidad Internacional Iberoamericana, Campeche, México; Universidad Internacional Iberoamericana, Campeche, México; Universidad de La Romana, La Romana, República Dominicana; Department of Clinical Laboratories Sciences, College of Applied Medical Sciences, Taif University, Taif 21944, Saudi Arabia; Department of Clinical Laboratories Sciences, College of Applied Medical Sciences, Taif University, Taif 21944, Saudi Arabia; Department of Clinical Laboratories Sciences, College of Applied Medical Sciences, Taif University, Taif 21944, Saudi Arabia; University of Valladolid, Valladolid, Spain; Department of Microbiology, Jahangirnagar University, Savar, Dhaka, Bangladesh

**Keywords:** Bangladesh, co-infection, COVID-19, dengue, epidemiology

## Abstract

**Background:**

Co-infection of dengue and COVID-19 has increased the health burden worldwide. We found a significant knowledge gap in epidemiology and risk factors of co-infection in Bangladesh.

**Methods:**

This study included 2458 participants from Dhaka city from 1 December 2021 to November 30 2023. We performed the Kruskal-Wallis test and χ^2^ test. Multivariable logistic regression was also performed.

**Results:**

Co-infection of dengue and COVID-19 was found among 31% of the participants. Coprevalence of dengue and COVID-19 was found in higher frequency in Jatrabari (14%) and Motijhil (11%). Severe (65%, *P* = .001) and very severe (78%, *P* = .005) symptoms were prevalent among the participants aged >50 years. Long-term illness was prevalent among the participants with co-infection (35%; 95% confidence interval [CI], 33–36) and COVID-19 (28%; 95% CI, 26–30). Co-infected participants had a higher frequency of heart damage (31.6%, *P* = .005), brain fog (22%, *P* = .03), and kidney damage (49.3%, *P* = .001). Fever (100%) was the most prevalent symptom followed by weakness (89.6%), chills (82.4%), fatigue (81.4%), headache (80.6%), feeling thirsty (76.3%), myalgia (75%), pressure in the chest (69.1%), and shortness of breath (68.3%), respectively. Area of residence (odds ratio [OR], 2.26; 95% CI, 1.96–2.49, *P* = .01), number of family members (OR, 1.45; 95% CI ,1.08–1.87; *P* <.001), and population density (OR, 2.43; 95% CI, 2.15–3.01; *P* = .001) were associated with higher odds of co-infection. We found that coinfected participants had a 4 times higher risk of developing severe health conditions (OR, 4.22; 95% CI, 4.11–4.67; *P* = .02).

**Conclusions:**

This is one of the early epidemiologic studies of co-infection of dengue and COVID-19 in Bangladesh.

## Teaser

Co-infection of dengue and COVID-19 has increased the global health burden. Co-prevalence of dengue and COVID-19 was greater in densely populated areas. Coinfected participants had a four times higher risk of developing severe health conditions (OR 4.22, 95% CI 4.11-4.67).

## Graphical Abstract

**Graphical Abstract:**
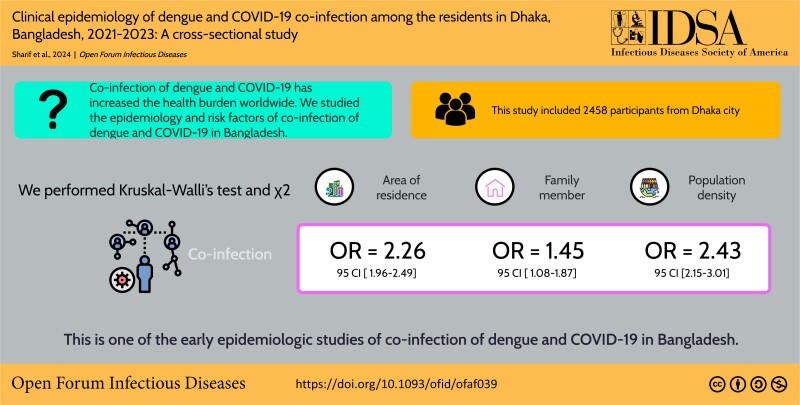
This graphical abstract is also available at Tidbit: https://tidbitapp.io/tidbits/clinical-epidemiology-of-dengue-and-covid-19-co-infection-among-the-residents-in-dhaka-bangladesh-2021-2023-a-cross-sectional-study?utm_campaign=tidbitlinkshare&utm_source=ITP

The COVID-19 pandemic has severely affected the people in Bangladesh since the first quarter of 2020 [1]. Almost 20 million cases and 30 000 deaths from COVID-19 have been documented from March 2020 to February 2024 in Bangladesh [1, 2]. Four distinct peaks of cases were identified during 2020 and 2023. They were confined between May 2020 and July 2020, March 2021 and May 2021, June 2021 and September 2021, and January 2022 and March 2022 [2–4]. Not only were daily cases and fatalities higher but the majority of cumulative cases and fatalities (∼80%) were recorded during these peaks [1, 3, 4].

About 350 million cases of dengue occur every year worldwide [5, 6]. Nearly half of the world's population (4 billion) is at risk of dengue virus infection [5–7]. Bangladesh is one of the major endemic regions of dengue outbreaks [5, 6]. In early 2000, dengue was reported first time in Bangladesh. Since 2000, outbreaks of dengue have been regularly documented in Bangladesh [6–8]. Instead of poor surveillance and underreporting, about 600 000 cases and 2600 deaths of dengue outbreaks have been confirmed in Bangladesh. Recently, in 2019 and 2023, larger outbreaks with about 101 500 and 321 073 cases were documented [5–9]. It is estimated that about two-thirds of the population will be at risk of dengue infection in the future [5, 9].

Though vaccines against COVID-19 were made available from February 2021 in Bangladesh, there are no effective vaccines against dengue [10–12]. Furthermore, the cocirculation of COVID-19 in dengue-endemic regions poses the greatest health threat among the people in Bangladesh. Dhaka (23° 45′ 50″ N, 90° 23′ 20″ E) is the capital and the most populous city in Bangladesh with about 100 000 persons/sq mi population density [13, 14]. Dhaka has remained the major hotspot of dengue outbreaks (80% of cases) in Bangladesh. The urbanization and centralization of administrations and health sectors have increased significantly in Dhaka after 2010. As a result, the density of breeding places of vectors of dengue virus has spiked contributing to larger outbreaks after 2019. After the COVID-19 outbreak, the majority of the cases were also documented in this region. However, there is a lack of studies on the co-prevalence and health risks of dengue and COVID-19 in Bangladesh. We designed this study to determine the epidemiology co-prevalence of dengue and COVID-19, associated factors, and health risks among the residents in Bangladesh.

## METHODS

### Ethical Approval

Ethical clearance for this study was taken from the Biosafety, Biosecurity & Ethical Committee at Jahangirnagar University, with an ethical approval number of BBEC, JU/M 2021/COVID-19/(8)1.

### Study Design and Sampling

This study was conducted using a cross-sectional design. Data were collected from different regions in Dhaka. The study spanned from 1 December 2021 to 30 November 2023. We found the majority of the cases of COVID-19 and a higher incidence of dengue in Dhaka during the study period compared to the previous period. Moreover, we covered the August to December (the prime time of dengue outbreaks) period for the 2 consecutive years. We used a structured questionnaire to collect data from the participants. We have collected data from individuals aged 10 years and older who tested positive for either COVID-19 or dengue and/or both. The study incorporated all participants, including sex, race, religion, occupation, and education levels, ensuring a reduction in potential biases. We enrolled the participants by using convenience sampling and invited them through online platforms. We included participants who tested dengue by NS1 antigen and/or immunoglobulin M/immunoglobulin G test (microplate-based enzyme-linked immunosorbent assay) and/or real-time reverse transcriptase polymerase chain reaction followed by the World Health Organization (WHO) recommendation and also COVID-19 by multiplex real-time reverse transcriptase polymerase chain reaction. Data acquisition encompassed both direct participants and hospitalized individuals. Informed consent was taken from all participants. We confined our study to local cases. A single response from each participant was counted as valid.

### Criteria for Inclusion and Exclusion

The inclusion criteria consisted of several parameters, including participants must be a local resident in Bangladesh, aged 10 years or older, infected by either COVID-19 or dengue or both, have not traveled outside the study areas during past 3 months, and tested for COVID-19 by WHO suggested tests. Exclusion criteria consisted of different factors such as participants not giving complete information on the questionnaire, aged younger than 10 years, not a local resident of Bangladesh, have taken any of the dose of COVID-19 vaccine from outside Bangladesh, traveled outside Dhaka within the past 3 months, were not confirmed for COVID-19 by a WHO suggested test method, and were not confirmed dengue positive by a WHO accepted method.

### Data Collection

The structured questionnaire was divided into 3 main sections. The first section included sociodemographic data including age, sex, occupation, residential location, educational background, prior infection history of dengue virus, previous health conditions, and travel history in the past 3 months. The second section focused on symptoms among the participants, duration of symptoms, severity, outcome, any fatality, treatment, or vaccination history. The third section included questions on co-infection, test methods, diagnosis center, treatment history, and vaccination history. Long-term illness was defined as persons with any symptoms associated with COVID-19 or dengue for 6 months or longer. The questionnaire was prepared in Bangla language and disseminated to the residents in Dhaka. The sample size was determined based on existing literature (n = 1258). However, our sample size was well above the calculated value. Before participation, objectives of the study were explained in detail to the participants.

### Statistical Analyses

The baseline data and responses in the demographic section were presented by using descriptive statistics such as mean, median, standard deviation, and interquartile range. We used the Kruskal-Wallis test for continuous variables. We also performed the χ^2^ test for the responses of categorical variables. For the identification of responsible factors with coprevalence of dengue and COVID-19 and clinical symptoms among the participants, we used a multivariable logistic regression model. We considered different factors in the multivariable model including the age of the participants (continuous variables with a class interval of 10 years), residence (insider or outsider) sex given at birth (male or female), subjective socioeconomic conditions, infection history, number of vaccine dose, type of diagnosis, preexisting medical conditions (both noncommunicable and infectious diseases and other health conditions), smoking history, education level, and blood grouping. We used another separate multivariable model to determine the associated factors responsible for severe and long-term health outcomes including very mild, mild, and moderate side effects versus severe or very severe by using previously described factors of the participants. We considered the findings statistically significant if *P* < .05. We used SAS version 9.4 (SAS Institute) and Microsoft Excel version 2023 (Microsoft, USA) for data analyses [5, 15].

## RESULTS

### Demographic Characteristics

This study included 2458 participants from Dhaka city (23°45′50″N 90°23′20″E) in Bangladesh. Among them, 100% were positive for COVID-19, followed by dengue (52%, 1278 of 2458), and both dengue and COVID-19 (31%, 763 of 2458), respectively. Male to female ratio was about 1:1 (1321:1137). The majority of the participants were aged between 20 and 29 years (28.2%, 692 of 2458) followed by 40–49 years (20.6%, 506 of 2458), 50–59 years (18.5%, 458 of 2458), and 30–39 years (16.7%, 410 of 2458), respectively (Table 1). About 48.7% of the participants were employed. The ratio of nonsmokers:smokers was 7:4. Preexisting health conditions were reported among 17.1%. The majority had moderate access to health services (48%, 1180 of 2458) followed by good (34.6%) and poor (17.4%), respectively. The ratio of participants from North:South cities was about 1:1. Among the participants, 52.5% (1290 of 2458) received COVID-19 vaccines. Oxford–AstraZeneca (49.4%) was the most prevalently given followed by Sinopharm BIBP (18.7%) and Pfizer-BioNTech (12.8%), respectively (Table 1).

**Table 1.** Demographic Characteristics of the Participants

Variables	COVID-19, N = 2458 (%)	Dengue, N = 1278 (%)	Both COVID-19 and Dengue, N = 763 (%)
**Sex**			
Male	1321 (53.7)	743 (58.1)	445 (58.3)
Female	1137 (46.3)	535 (41.9)	318 (41.7)
**Age groups, y**			
10–19	145 (5.9)	312 (24.4)	126 (16.5)
20–29	692 (28.2)	226 (17.7)	214 (23.7)
30–39	410 (16.7)	214 (16.7)	134 (17.6)
40–49	506 (20.6)	269 (21)	154 (20.2)
50–59	454 (18.5)	118 (9.2)	89 (11.7)
60–69	208 (8.5)	108 (8.5)	75 (9.8)
>70	43 (1.7)	31 (2.4)	4 (0.5)
**Occupation**			
Student	657 (26.7)	517 (40.5)	204 (26.7)
Employed	1196 (48.7)	632 (49.5)	395 (51.8)
Unemployed	605 (24.6)	129 (10.1)	164 (21.5)
**Smoking history**			
Smoker	865 (35.2)	383 (30)	252 (33)
Nonsmoker	1593 (64.8)	895 (70)	511 (67)
**Health status**			
Preexisting health conditions	421 (17.1)	213 (16.7)	165 (21.6)
Hospitalization	102 (4.1)	53 (4.1)	65 (8.5)
ICU admission	87 (3.5)	38 (3)	29 (3.8)
**Residence**			
Dhaka North City Corporation	1181 (48)	643 (50.3)	466 (61.1)
Dhaka South City Corporation	1277 (52)	635 (49.7)	297 (38.9)
**Education**	…	…	…
Under SSC	347 (14.1)	384 (30)	101 (13.2)
SSC-HSC	1453 (59.1)	521 (40.8)	272 (35.6)
HCS-graduate	658 (26.8)	373 (29.2)	390 (51.1)
**Access to health services**			
Poor	428 (17.4)	231 (17.2)	124 (13.4)
Moderate	1180 (48)	678 (50.6)	287 (31.1)
Good	850 (34.6)	432 (32.2)	512 (55.5)
**COVID-19 vaccination**			
Yes	1290 (52.5)	784 (61.3)	465 (60.9)
No	1168 (47.5)	494 (38.7)	298 (39.1)
**COVID-19 vaccination**			
Oxford–AstraZeneca	637 (49.4)	265 (33.8)	195 (41.9)
Sinopharm BIBP	241 (18.7)	146 (18.6)	122 (26.2)
Pfizer-BioNTech	165 (12.8)	142 (18.1)	82 (17.6)
Sinovac	132 (10.2)	121 (15.4)	42 (9)
Moderna	115 (8.9)	110 (14)	24 (5.2)

Abbreviations: ICU, intensive care unit.

### Temporal and Spatial Distribution of Dengue and COVID-19

**Figure 1.**
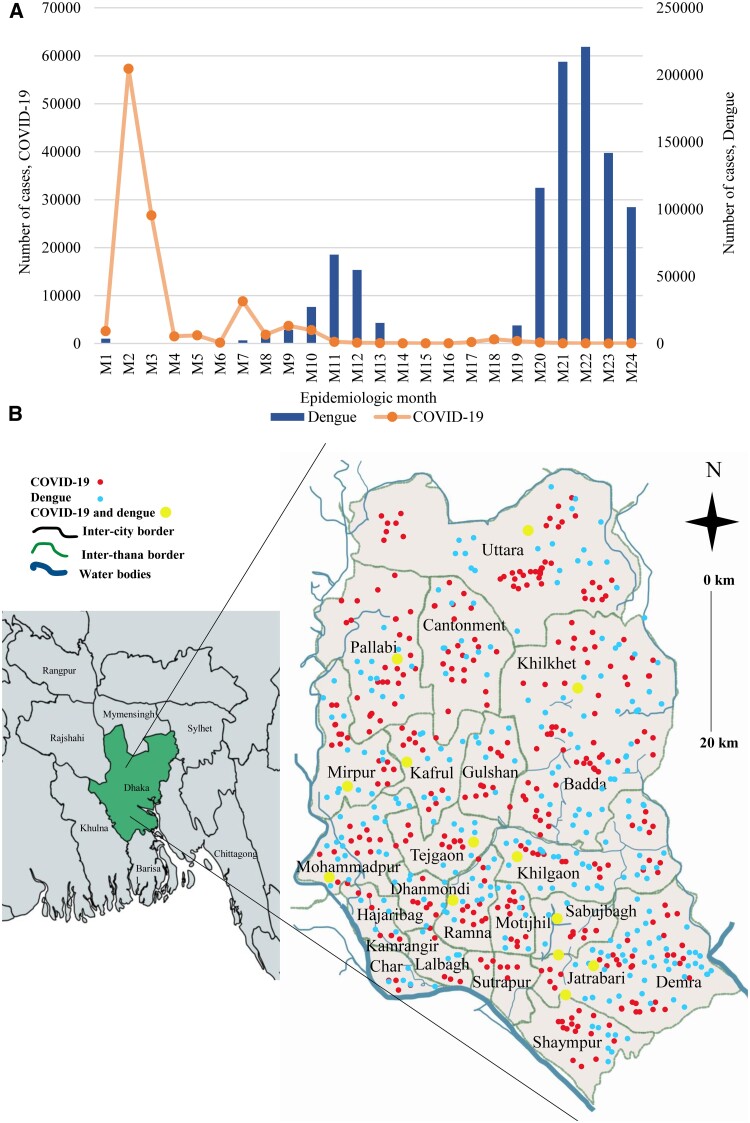
(*A*) Temporal distribution of cases of COVID-19 and dengue. (*B*) Maps of the incidence of COVID-19, dengue, and co-infection across Dhaka city.

Cases of COVID-19 were the most prevalent during epidemiologic month 1 (M1) and month 4 (M4) in Dhaka city. The highest number of cases of COVID-19 was recorded in M2 (58 000 cases), followed by M3 (27 000 cases), M7 (10 000 cases), and M9 (5000 cases), respectively. The number of cases remained <5000 during M10 and M24. On the other hand, 2 distinct peaks of dengue cases were noticed. One peak was apparently confined from M10 to M13 and another within M20 through M24. The highest number of cases was documented in M22 (63 000 cases) followed by M21 (57 000 cases), M23 (40 000 cases), and M20 (33 000 cases), respectively. Incidence of dengue and COVID-19 increased simultaneously in M8, M9, and M10 (Figure 1*A*). The mean number co-infection cases was significant from July to December (*P* <.05) of the study period (Supplementary Table I). Cases of co-infection were widely distributed across Dhaka city. The prevalence of dengue was higher in the south city corporation areas (65%) and COVID-19 in the north city corporation areas (58%). Coprevalence of dengue and COVID-19 was found in higher frequency in Jatrabari (14%), Motijhil (11%), Khilgaon (10%), Tejgaon (9%), Dhanmondi (9%), Mohammadpur (8%), Mirpur (7%), Kafrul (7%), Khilkhet (5%), Pallabi (5%), and Uttara (5%) (Figure 1*B*).

### Severity and Duration of Signs and Symptoms

**Figure 2.**
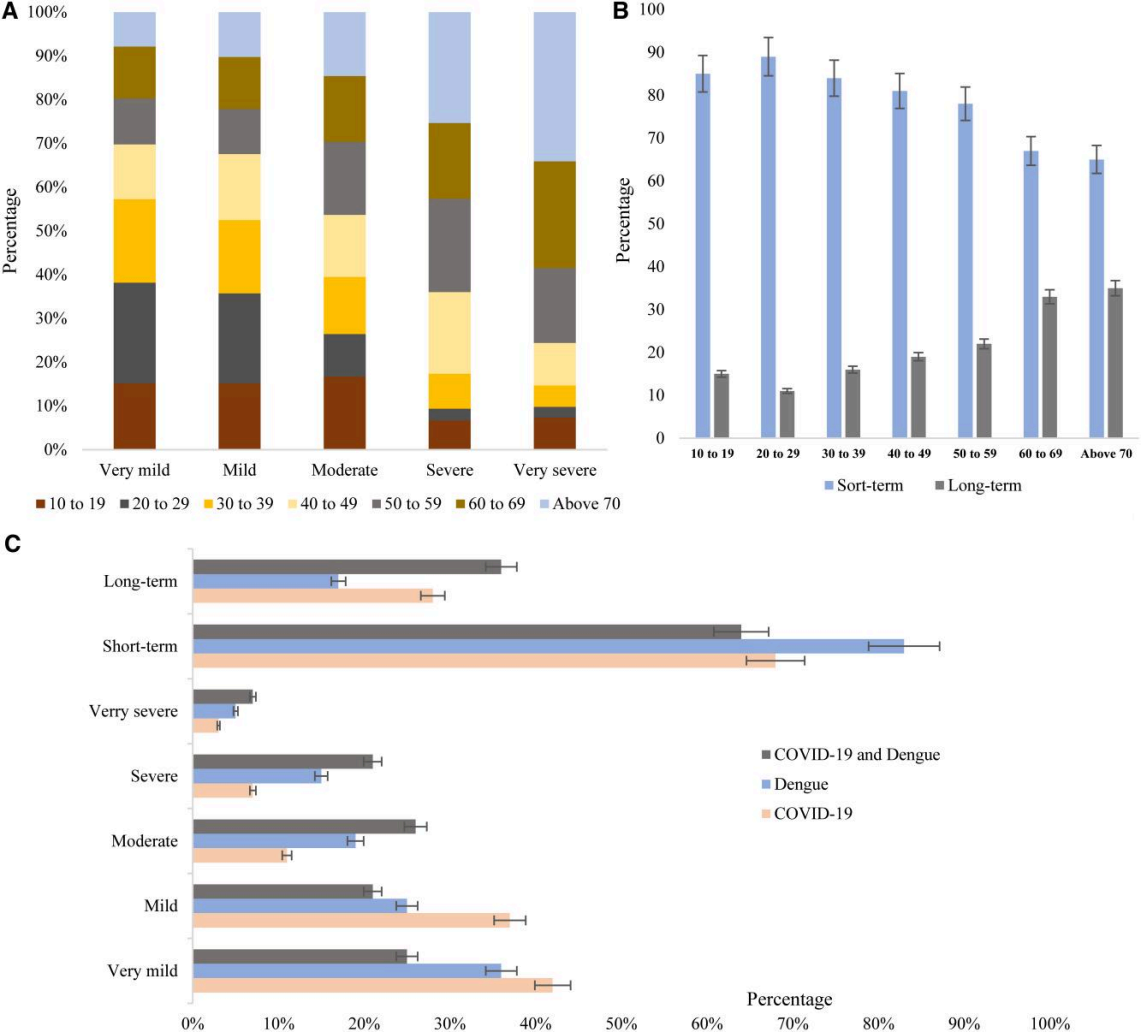
(*A*) Age distribution of severity of symptoms, (*B*) age distribution of duration of symptoms, and (*C*) percentage of severity and duration of symptoms among the participants.

The severity of clinical signs and symptoms was defined and categorized into 5 groups: very mild, mild, moderate, severe, and very severe. Based on duration, we categorized symptoms into short-term and long-term. The severity of symptoms varied among different age groups. Severe (65%, *P* = .001) and very severe (78%, *P* = .005) symptoms were prevalent among the participants aged 50 years or older, and very mild (70%, *P* = .05), mild (67%, *P* = .005), and moderate (55%, *P* = .001) symptoms were frequent among the participants aged 10 to 40 years (Figure 2*A*). Short-term symptoms were found in the highest frequency (68%–95%, *P* = .01) followed by long-term (10%–30%, *P* = .05). Long-term symptoms lasting for more than 2 weeks were found among 19%–36% (*P* = .001) of the participants aged >40 years (Figure 2*B*).

Participants with COVID-19 infection experienced very mild symptoms (42%; 95% confidence interval [CI], 40–44) in the highest frequency followed by mild (37%; 95% CI, 35–39), moderate (10%; 95% CI, 9–11), severe (8%; 95% CI, 7–9), and very severe (3%; 95% CI, 1–4), respectively. Severe conditions were found among 15% (95% CI, 14–16) and very severe among 5% (95% CI, 4–6) of the dengue-positive participants. Severe (20%; 95% CI, 19–22) and very severe (8%; 95% CI, 7–10) symptoms were documented in the highest frequency among the participants with both COVID-19 and dengue infection (Figure 2*C*). Acute illness was prevalent among participants with dengue (83%; 95% CI, 80–90) followed by COVID-19 (68%; 95% CI, 65–72) and co-infection (63%; 95% CI, 58–66). Long-term illness was prevalent among the participants with co-infection (35%; 95% CI, 33–36) and COVID-19 (28%; 95% CI, 26–30) (Figure 2*C*).

### Prevalence and Association of Symptoms

#### Both COVID-19 and Dengue Positive

Among the co-infected participants, fever (100%) was the most prevalent symptoms followed by weakness (89.6%), chills (82.4%), fatigue (81.4%), headache (80.6%), feeling thirsty (76.3%), myalgia (75%), pressure in the chest (69.1%), shortness of breath (68.3%), sleeping difficulty (56.1%), loss of smell and test (55.2%), sore throat (54.1%), kidney damage (49.3%), pale skin (49.5%), rapid breathing (49.1%), nausea (46.8%), vomiting (46.1%), and abdominal pain (46.8%), respectively. Co-infection with COVID-19 and dengue was significantly associated (*P* = <.05) with onset or continuation of symptoms. Co-infected participants had higher frequency of heart damage (31.6%, *P* = .005), brain fog (22%, *P* = .03), kidney damage (49.3%, *P* = .001), liver damage (14.3%, *P* = .05), hemorrhage (42.3%, *P* <.001), fluid accumulation (22.9%, *P* = .001), mucosal bleeding (46.4%, *P* = .04), leukopenia (33.7%, *P* = .005), and gastrointestinal bleeding (33.7%, *P* = .04) than monoinfected by either dengue or COVID-19 (Table 2). Fever with high temperature (104.1–106.0) and lasting >14 days were most prevalent among the co-infected participants (37.9% and 35.3%, respectively).

**Table 2.** Association of Symptoms With Co-infection of Dengue and COVID-19

Side Effects, N (%)	COVID-19	Dengue	Both COVID-19 and Dengue	*P V*alue
	N = 2458 (%)	N = 1278 (%)	N = 763 (%)	
Fever (°F)	1653 (73.8)	1273 (99.6)	763 (100)	<.001
Mild/low-grade fever (100.5–102.2)	821 (49.7)	432 (33.9)	247 (32.4)	.05
Moderate-grade fever (102.2–104.0)	532 (32.2)	321 (25.2)	164 (21.5)	.01
High-grade fever (104.1–106.0)	263 (15.9)	314 (24.7)	289 (37.9)	.005
Hyperpyrexia (>106.0)	37 (2.2)	206 (16.2)	63 (8.3)	.003
Duration of fever				
<7 d	524 (31.7)	358 (28.1)	160 (21)	.005
7 to <14 d	957 (57.9)	764 (60)	334 (43.8)	.01
>14 d	172 (10.4)	156 (11.9)	269 (35.3)	.04
Cough	1843 (75)	641 (50.2)	538 (70.5)	.005
Headache	2156 (87.7)	1054 (82.5)	615 (80.6)	<.001
Retroorbital pain	757 (30.8)	364 (28.5)	303 (39.7)	.001
Muscle and joint pain	468 (19)	981 (76.8)	315 (41.3)	<.001
Myalgia	735 (29.9)	845 (66.1)	572 (75)	<.001
Fatigue	1598 (65)	954 (74.6)	621 (81.4)	.001
Swollen glands	189 (7.7)	792 (62)	153 (20.1)	<.001
Sore throat	1643 (66.8)	214 (16.7)	413 (54.1)	.05
Congestion or runny nose	1267 (51.5)	313 (24.5)	256 (33.6)	.03
New loss of taste or smell	1196 (48.7)	132 (10.3)	421 (55.2)	.001
Vomiting	452 (18.4)	226 (17.7)	352 (46.1)	.001
Chills	1511 (61.5)	985 (77.1)	629 (82.4)	.05
Shortness of breath	655 (26.6)	378 (29.6)	521 (68.3)	.001
Nausea	1154 (46.9)	348 (27.2)	357 (46.8)	.002
Vomiting	687 (27.9)	145 (11.3)	352 (46.1)	.001
Severe abdominal pain	254 (10.3)	326 (25.5)	357 (46.8)	.05
Persistent vomiting	178 (7.2)	58 (4.5)	149 (19.5)	.001
Rapid breathing	534 (21.7)	106 (8.3)	375 (49.1)	<.001
Bleeding gums or nose	86 (3.5)	346 (27.1)	217 (28.4)	<.005
Restlessness	656 (26.7)	385 (30.1)	219 (28.7)	.002
Blood in vomit or stool	65 (2.6)	346 (27.1)	178 (23.3)	.001
Diarrhea	372 (15.1)	215 (16.8)	278 (36.4)	.05
Being very thirsty	1056 (43)	647 (50.6)	582 (76.3)	.005
Pale and cold skin	343 (14)	436 (34.1)	378 (49.5)	<.001
Feeling weak	1871 (76.1)	1062 (83.1)	684 (89.6)	.001
Persistent pain or pressure in the chest	1534 (62.4)	321 (25.1)	527 (69.1)	.03
Inability to wake or stay awake	732 (29.8)	265 (20.7)	543 (21.2)	.02
Difficulty sleeping	621 (25.3)	287 (22.5)	428 (56.1)	.05
Appetite loss	1093 (44.5)	375 (29.3)	264 (34.6)	.001
Rash	145 (5.9)	932 (72.9)	323 (42.3)	<.001
Brain fog	218 (8.9)	41 (3.2)	168 (22)	.03
Kidney damage	587 (23.9)	154 (12.1)	376 (49.3)	.001
Heart damage	254 (10.3)	23 (1.8)	241 (31.6)	.005
Liver damage	156 (6.3)	58 (4.5)	109 (14.3)	.05
Hemorrhage	71 (2.9)	523 (40.9)	323 (42.3)	<.001
Increased blood glucose	489 (19.9)	53 (4.1)	286 (37.5)	.05
Clinical fluid accumulation	131 (5.3)	153 (12)	175 (22.9)	.001
Mucosal bleed	15 (0.6)	433 (33.9)	354 (46.4)	.04
Liver enlargement	159 (6.5)	237 (18.5)	210 (27.5)	.05
Leukopenia	143 (5.8)	461 (36.1)	257 (33.7)	.005
Skin bleeding	0	437 (34.2)	215 (28.2)	.001
Gastrointestinal bleeding	4 (0.2)	362 (28.3)	257 (33.7)	.04

*P* value <.05 was considered significant.

#### COVID-19 Positive

Participants with COVID-19 infection reported different signs and symptoms. Among them, headache (87.7%) was the most prevalent, followed by weakness (76.1%), cough (75%), fever (73.8%), sore throat (66.8%), fatigue (65%), chills (61.5%), chest pain (62.4%), and runny nose (51.5%), respectively. The majority of the participants reported mild/low-grade fever (49.7%) followed by moderate-grade fever (32.2%) lasting for 7 to <14 days. Kidney damage (23.9%) and increased blood glucose level (19.9%) was reported significantly among other symptoms. A significant association was found between COVID-19 infection and the development of symptoms (*P* < .05).

#### Dengue Positive

Among the dengue-positive participants, fever (99.6%) was the most prevalent followed by weakness (82.5%), headache (82.5%), chills (77.1%), muscle and joint pain (76.8%), fatigue (74.6%), rash (72.9%), myalgia (66.1%), swollen glands (62%), cough (50.2%), and feeling thirsty (50.6%), respectively. Hemorrhage (40.9%), leukopenia (36.1%), skin bleeding (34.2%), mucosal bleeding (33.9%), and gastrointestinal bleeding (28.3%) were more frequent among dengue patients than others.

#### Risk Factors of Co-infection of Dengue and COVID-19

A multivariable logistic regression model was built to determine the risk factors of co-infection among the participants. Among different demographic factors, age (OR, 1.42; 95% CI ,1.25–2.02; *P* = .01), sex-female (OR, 1.67; 95% CI, 1.26–1.91; *P* = .01), blood group (OR, 1.25; 95% CI, 1.03–1.63; *P* = .003), and smoking habit-smokers (OR, 1.55; 95% CI, 1.11–1.82; *P* = .001) had significantly higher odds of co-infection by dengue and COVID-19. Area of residence (OR, 2.26; 95% CI, 1.96–2.49; *P* = .01), frequent movements within the city (OR, 2.16; 95% CI, 1.85–2.64; *P* = <.005), number of family members (OR, 1.45; 95% CI, 1.08–1.87; *P* <.001), and population density (OR, 2.43; 95% CI, 2.15–3.01; *P* = .001) were associated with higher odds of co-infection. Dengue-infected participants (OR, 4.12; 95% CI, 4.05–4.86; *P* = .05) had the highest odds of co-infection followed by COVID-19 infection (OR, 3.49; 95% CI; 3.25–4.01; *P* = .005) and presence of infected family member within the same house (OR, 2.68; 95% CI, 2.25–3.12; *P* = .002). Among the preexisting comorbidities, diabetes (OR, 1.94; 95% CI, 1.43–2.25; *P* = .01), influenza (OR, 1.36; 95% CI, 1.02–1.53; *P* = .005), HIV (OR, 1.43; 95% CI, 1.13–1.76; *P* = .001), and chronic obstructive pulmonary disease (OR, 1.26; 95% CI, 1.06–1.64; *P* = .001) were significantly associated with higher odds of co-infection (Table 3).

**Table 3.** Odds of Co-infection Among the Residents in Dhaka

Characteristics	OR (95% Confidence Interval)	*P* Value
COVID-19 infection (positive vs negative)	3.49 (3.25–4.01)	.005
Dengue infection (positive vs negative)	4.12 (4.05–4.86)	.05
Age (per 10 y)	1.42 (1.25–2.02)	.01
Sex (female vs male)	1.67 (1.26–1.91)	.01
Occupation	0.52 (0.25–0.96)	.001
Smoking habit	1.55 (1.11–1.82)	.001
Education level	1.28 (1.05–1.75)	.02
Blood group	1.25 (1.03–1.63)	.003
Health status (Healthy vs comorbid)	0.34 (0.15–0.82)	.05
Residence area	2.26 (1.96–2.49)	.01
Previous infection of dengue	2.65 (2.06–2.91)	.001
Use of mosquito net	0.35 (0.09–0.75)	.005
Use of mosquito repellents	0.78 (0.41–0.96)	.001
Family member number	1.45 (1.08–1.87)	<.001
Use of face masks	0.32 (0.10–0.88)	.005
Use of hand sanitizers	0.54 (0.26–0.97)	.05
Hand cleaning	0.62 (0.23–0.95)	.01
Population density	2.43 (2.15–3.01)	.001
Movements within the city	2.16 (1.85–2.64)	<.005
Diagnostic centers near residence	1.23 (1.01–1.65)	.003
Infected family member	2.68 (2.25–3.12)	.002
COVID-19 vaccination (yes vs no)	1.21 (1.02–1.83)	.001
Homogenous doses	1.02 (0.81–1.42)	.005
Heterogenous doses	1.25 (1.03–1.54)	.05
Diabetes	1.94 (1.43–2.25)	.01
CVDs	0.65 (0.14–0.82)	.001
Hypertension	0.69 (0.13–0.96)	.02
Autoimmune diseases	0.43 (0.14–0.85)	.03
Influenza	1.36 (1.02–1.53)	.005
HIV	1.43 (1.13–1.76)	.001
Asthma	0.43 (0.14–0.65)	.03
Anemia	0.35 (0.13–0.64)	.05
COPD	1.26 (1.06–1.64)	0.001

*P* value <.05 was considered significant.

Abbreviations: COPD, chronic obstructive pulmonary disease; CVD, cardiovascular disease; OR, odds ratio.

#### Risk of Severe and Long-term Health Effects

Risk factors for both severe and long-term symptoms were determined. We found that coinfected participants had a 4 times higher risk of developing severe health conditions (OR, 4.22; 95% CI, 4.11–4.67; *P* = .02) followed by COVID-19 infection (OR, 4.15; 95% CI, 3.98–4.65; *P* = .005), dengue infection (OR, 3.67; 95% CI, 3.29–3.94; *P* = .005), previous comorbidities (OR, 3.68; 95% CI, 3.43–3.88; *P* = .05), hypertension (OR, 2.56; 95% CI, 2.25–2.73; *P* = .001), participants requiring hospitalization (OR, 2.53; 95% CI, 2.24–2.85; *P* = .005), or fever (OR, 2.31; 95% CI, 2.14–2.69; *P* < .001), respectively. Preexisting comorbidities including autoimmune diseases (OR, 1.83; 95% CI, 1.52–2.24; *P* = .001), asthma (OR, 1.75; 95% CI, 1.42–1.95; *P* = .004), tuberculosis (OR, 1.86; 95% CI, 1.65–2.21; *P* = .001), cardiovascular diseases (OR, 1.67; 95% CI, 1.42–1.98; *P* = .002), diabetes (OR, 1.47; 95% CI, 1.22–1.85; *P* = .01), HIV (OR, 1.56; 95% CI, 1.23–1.94; *P* = .001), and anemia (OR, 1.45; 95% CI, 1.16–1.74; *P* = .001) were associated with higher odds of long-term symptoms. We also found that COVID-19 vaccination (OR, 1.35; 95% CI, 1.06–1.87; *P* = .001) and participants with symptoms of leukopenia (OR, 1.28; 95% CI, 1.05–1.57; *P* = .05), breathing problems (OR, 1.64; 95% CI, 1.35–1.87; *P* = .05), bleeding gums (OR, 1.87; 95% CI, 1.54–2.21; *P* = .01), and hemorrhage (OR, 1.54; 95% CI, 1.25–1.63; *P* = .01) suffered from severe health outcomes (Table 4).

**Table 4.** Odds of Severe and Long-term Symptoms Among the Participants

Characteristics	OR (95% Confidence Interval)	*P* Value
COVID-19 infection (positive vs negative)	4.15 (3.98–4.65)	.005
Dengue infection (positive vs negative)	3.67 (3.29–3.94)	.01
Coinfection	4.22 (4.11–4.67)	.02
Age (per 10 y)	1.45 (1.21–1.78)	.005
Sex (male vs female)	1.84 (1.68–2.23)	.05
Occupation	0.62 (0.35–0.95)	.03
Smoking habit	1.87 (1.58–2.16)	.001
Health status (comorbid vs healthy)	3.68 (3.43–3.88)	.05
Residence	1.36 (1.21–1.53)	.06
Education level	1.21 (1.01–1.65)	.001
Blood group	1.43 (1.16–1.85)	.005
Access to health services	1.46 (1.21–1.73)	<.001
COVID-19 vaccination	1.35 (1.06–1.87)	.001
Single dose	1.16 (1.03–1.71)	<.001
Double dose	1.34 (1.15–1.58)	.05
Triple dose	1.49 (1.23–1.95)	.001
Homogenous dose	1.15 (0.92–1.69)	<.001
Heterogenous dose	1.17 (0.92–1.54)	.002
Hospitalization	2.53 (2.24–2.85)	.005
Hemorrhage	1.54 (1.25–1.63)	.01
Duration of fever	2.31 (2.14–2.69)	<.001
Shortness of breath	1.64 (1.35–1.87)	.05
Blood in vomit or stool	1.43 (1.06–1.73)	.01
Persistent pain or pressure in the chest	1.47 (1.23–1.89)	<.001
Bleeding skin or gum	1.87 (1.54–2.21)	.01
Leukopenia	1.28 (1.05–1.57)	.05
Autoimmune diseases	1.83 (1.52–2.24)	.001
Long COVID-19	1.43 (1.21–1.76)	<.001
Diabetes	1.47 (1.22–1.85)	.01
CVDs	1.67 (1.42–1.98)	.002
Hypertension	2.56 (2.25–2.73)	.001
Influenza	1.43 (1.25–1.86)	.05
HIV	1.56 (1.23–1.94)	.001
Asthma	1.75 (1.42–1.95)	.004
Anemia	1.45 (1.16–1.74)	.001
COPD	1.78 (1.39–1.98)	.05
Tuberculosis	1.86 (1.65–2.21)	.001
Bronchitis	1.32 (1.05–1.67)	.001

*P* value <.05 was considered significant.

Abbreviations: COPD, chronic obstructive pulmonary disease; CVD, cardiovascular disease; OR, odds ratio.

## DISCUSSION

Cocirculation of COVID-19 and dengue during 2021 and 2023 have created a major health burden worldwide [15–21]. Co-infection by COVID-19 and dengue have been involved in worse health outcomes including higher mortality and prolonged morbidity [22, 23]. To the best of our knowledge, before this study, no other research was conducted on epidemiology and risks of co-infection of these diseases among the study participants in Bangladesh. Our study found 4 major aspects of epidemiology, risk, and clinical outcomes of coprevalence of dengue and COVID-19. First, we found a higher incidence of co-infection (31%) among the residents in Dhaka city. Dhaka is the most populous capital city in Bangladesh and remains the main focal place of dengue outbreaks, with >70% of cases in Bangladesh. After the onset of COVID-19, residents of the capital were mostly affected. Cocirculation of both COVID-19 and dengue from 2021 and 2023 was involved in a higher incidence of co-infection among the city dwellers. This is a newer finding compared to previous studies on COVID-19 and dengue in Bangladesh [24, 25]. However, we found a higher incidence of dengue (52%) compared to previous studies in Bangladesh and nearby countries [5–7]. Co-infection was prevalent in males (58%) and participants aged <40 years (61%). These findings are in good agreement with previous studies [5, 7]. Second, we found that during the COVID-19 peak, cases of dengue were lowest. However, after the reduction of cases of COVID-19 and the removal of restrictions regarding COVID-19, cases of dengue increased gradually and became the highest during the last 5 epidemiological weeks. The prevalence of co-infection was distributed equally throughout the study. These findings strongly supported that during the COVID-19 pandemic, cases of dengue were underreported. Our data are similar to recent studies conducted in different countries in South and Southeast Asia [15–21]. Cases of dengue and COVID-19 were widely distributed throughout the Dhaka city. However, we found the incidence of co-infection was significantly higher in the south city corporation areas, including Jatrabari, Motijhil, Dhanmondi, and Mohammadpur. Along with high population density, the presence of diagnostic centers, hospitals, and clinics near the residences of the participants also contributed to the higher prevalence of dengue in the south city corporation areas. A higher population density had a significant effect on the transmission of both the COVID-19 and dengue outbreaks. Previous studies in Bangladesh, Japan, and Saudi Arabia reported the association of higher population density with increased transmission of the SARS-CoV-2 virus [26, 27]. Similarly, the transmission cycle of the dengue virus is easier to maintain in highly populated areas and families with a lot of members. One probable reason might be the availability of hosts become easier in populated areas.

Third, we found co-infection was more significantly associated (*P* < .05) with severe and long-term symptoms among the residents than monoinfection by either COVID-19 or dengue. The severity and duration of symptoms were also age-dependent. Increased incidence of severe (∼65%) to very severe (∼80%) health conditions was observed among people aged older than 50 years. Similarly, long-term symptoms gradually increased from 10% to 36% among the participants aged 20 years to older than 70 years, respectively. Prolonged morbidity increased the social and economic burdens of the people. These findings suggest that the health burden among the residents in Dhaka city was intensified by the cocirculation of both dengue and COVID-19. Furthermore, damage in the major organs involving heart (31%), kidney (49%), and liver (14%) were significantly associated with co-infection (*P* < .05). Infection with dengue virus and SARS-CoV-2 at the same time has a profound impact on the immune system and may have an impact on intensified damage to internal organs. Additionally, the pathogenesis and cell destruction of both of the viruses among the co-infected participants were probably more effective than singly infected individuals. These findings need further molecular immunological studies to reveal the exact mechanism of increased severity of disease among the co-infected individuals. Some exclusive symptoms of dengue including internal bleeding and bleeding from skin, gum, and leukopenia also increased among the co-infected participants. These findings are supported by previous studies and need more specific investigation in the future [28–34]. Fourth, this study reported that increased risk of co-infection was associated with demographic factors, social factors, and previous health status. Participants with previous infections of dengue, COVID-19, HIV, asthma, and diabetes had higher odds of co-infection (*P*-value <.05). Among social factors, population density and residential areas had an increased association with the risk of co-infection. These findings are relatively new in Bangladesh [24, 25, 33, 35]. On the other hand, we found that the use of mosquito repellents and, or nets, hand sanitizers, and face masks was associated (*P* < .05) with lower odds of co-infection. These findings are in good agreement with previous studies conducted on either dengue or COVID-19 outbreaks in Bangladesh [35, 36]. We also found that the odds of severe outcomes among the participants with co-infection increased approximately 4 times (*P* = .02). Male participants with comorbidities and smoking habits had significantly (*P* <05) higher odds of both severe and long-term health effects. Several previous studies in Bangladesh and nearby countries, including India, Pakistan, and Sri Lanka, support these findings [37–39].

This study's findings can significantly supplement the development of revised clinical guidelines and contribute to improved healthcare practices in Bangladesh and other lower-middle-income countries. In dengue-endemic regions, healthcare providers should carefully observe their patients for any extended symptoms of co-infection by other viruses and maintain respiratory etiquette and hand hygiene in the facilities. Furthermore, the establishment of a dedicated department for the diagnosis, treatment, and management of coinfection can contribute to the reduction of health burden. Because the incidence of coinfection is associated with a higher risk of severe and prolonged health outcomes, proper policies and guidelines to detect and control the coinfection should be developed locally and internationally. Along with seasonal management, long-term guidelines for treatment and prevention can also contribute to the mitigation of health burdens in Bangladesh.

Despite large samples and robust analyses, this study has several limitations. We have self-reported data from the participants; as a result, we could not include details of clinical diagnostic reports. Further, because of the lack of these data, we could not include the mechanism of severe health effects among the participants with co-infection. As we could not follow the participants for a long time, we used a cross-sectional study resulting in limitations in establishing causality. In addition, there might be some recall biases from the participants, which can be improved in a longitudinal study in the future. One of the major challenges is the proper identification of cocirculating viruses in resource-limited settings like Bangladesh. Because of the overlapping symptoms, the majority of people and healthcare providers limit the diagnosis to a single pathogen. Hence, they undermine the diversity of actual pathogens. In this study, we focused on 2 viruses, which can be increased in future studies. However, the findings can be used as baseline data to initiate future longitudinal studies on co-infection. In the future, the inclusion of data from histopathological tests could give more accurate predictions associated with co-infection. On the other hand, the main strength of this study was the inclusion of a large number of participants, including the major peaks of COVID-19 outbreaks, including the prime season of dengue outbreaks in the study period, and performing appropriate statistical analysis.

## CONCLUSION

This study reported a high prevalence (31%) of co-infection of dengue and COVID-19 among the residents in Dhaka city. Further, we reported that along with age and sex, the presence of comorbidities and residential areas, population density, preventive practices, and movement inside the city have significant influence on co-infection. The risk of prolonged morbidity increased significantly among the co-infected people. This is one of the first reports of epidemiology and risk analysis of co-infection of SARS-CoV-2 and dengue viruses in Bangladesh. The findings of this study highlighted that the general people, health practitioners, and policymakers should take more robust and specific steps in identifying the accurate pathogens with overlapping symptoms in dengue-endemic areas. This study created a comprehensive insight into the epidemiology and risk factors of cooccurrence of COVID-19 and dengue and will aid in policy-making to reduce the health burden in the future.

## Supplementary Material

ofaf039_Supplementary_Data

## Supplementary Data

Supplementary materials are available at *Open Forum Infectious Diseases* online. Consisting of data provided by the authors to benefit the reader, the posted materials are not copyedited and are the sole responsibility of the authors, so questions or comments should be addressed to the corresponding author.

## Notes

***Acknowledgments.*** The authors extend their appreciation to Taif University, Saudi Arabia, for supporting this work.

***Author contributions*.** N.S. and S.K.D. developed the study design; R.R.O., N.S., and T.S. conducted microbiological tests; N.S., A.K., R.R.O., F.M.A., M.A.A., and K.J.A. performed data analysis; N.S., A.K., and R.R.O. wrote the manuscript; N.S., R.S.R.S., E.G.V., and S.K.D. reviewed the manuscript; N.S. and S.K.D. supervised the work. All authors have read and approved the final version of the manuscript.

***Availability of data and materials.*** All the data are available in this manuscript.

***Financial support*.** This research was partially funded by Taif University, Taif, Saudi Arabia (TU-DSPP-2024-05).
