# Supplementary material for: Clinical Epidemiology of Dengue and COVID-19 Co-infection Among the Residents in Dhaka, Bangladesh, 2021–2023: A Cross-sectional Study
Source: Open Forum Infect Dis. 2025 Jan 25;12(2):ofaf039. doi: 10.1093/ofid/ofaf039 (PMC11839405; doi:10.1093/ofid/ofaf039)
Supplement: ofaf039_Supplementary_Data [file ofaf039_supplementary_data.docx]

**Supplementary Table I.** Seasonality of co-infection between SARS-CoV-2 and dengue viruses. Comparison of positive cases (mean value) of single infection and co-infection. Mann-Whitney U test was performed. A *p*-value of less than 0.05 was considered as significant.

| **Month** | **Single infection (*p*-value)** | **Co-infection (*p*-value)** |
| --- | --- | --- |
| January vs. February | 0.04 | 0.31 |
| February vs. March | 0.08 | 0.92 |
| March vs. April | 0.03 | 0.41 |
| April vs. May | 0.001 | 0.08 |
| May vs. June | 0.05 | 0.06 |
| June vs. July | 0.23 | 0.25 |
| July vs. August | 0.001 | 0.005 |
| August vs. September | 0.003 | 0.001 |
| September vs. October | 0.005 | <0.001 |
| October vs. November | 0.05 | 0.005 |
| November vs. December | 0.001 | 0.05 |
| December vs January | 0.05 | 0.001 |
